# High-Quality Recrystallization of Amorphous Silicon on Si (100) Induced via Laser Annealing at the Nanoscale

**DOI:** 10.3390/nano13121867

**Published:** 2023-06-15

**Authors:** Zhuo Chen, Huilong Zhu, Guilei Wang, Qi Wang, Zhongrui Xiao, Yongkui Zhang, Jinbiao Liu, Shunshun Lu, Yong Du, Jiahan Yu, Wenjuan Xiong, Zhenzhen Kong, Anyan Du, Zijin Yan, Yantong Zheng

**Affiliations:** 1 Key Laboratory of Microelectronics Devices & Integrated Technology, Institute of Microelectronics, Chinese Academy of Sciences, Beijing 100029, China; chenzhuo@ime.ac.cn (Z.C.); wangqi@ime.ac.cn (Q.W.); xiaozhongrui@ime.ac.cn (Z.X.); zhangyongkui@ime.ac.cn (Y.Z.); liujinbiao@ime.ac.cn (J.L.); lushunshun@ime.ac.cn (S.L.); duyong@ime.ac.cn (Y.D.); yujiahan@ime.ac.cn (J.Y.); xiongwenjuan@ime.ac.cn (W.X.); kongzhenzhen@ime.ac.cn (Z.K.); duanyan@ime.ac.cn (A.D.); yanzijin@ime.ac.cn (Z.Y.); zhengyantong@ime.ac.cn (Y.Z.); 2 Microelectronics Institute, University of Chinese Academy of Sciences, Beijing 100049, China; 3 Process Integration, Beijing Superstring Academy of Memory Technology, Beijing 100176, China; guilei.wang@bjsamt.org.cn

**Keywords:** vertical nanosheet, laser annealing, recrystallization, Si cap

## Abstract

At sub-3 nm nodes, the scaling of lateral devices represented by a fin field-effect transistor (FinFET) and gate-all-around field effect transistors (GAAFET) faces increasing technical challenges. At the same time, the development of vertical devices in the three-dimensional direction has excellent potential for scaling. However, existing vertical devices face two technical challenges: “self-alignment of gate and channel” and “precise gate length control”. A recrystallization-based vertical C-shaped-channel nanosheet field effect transistor (RC-VCNFET) was proposed, and related process modules were developed. The vertical nanosheet with an “exposed top” structure was successfully fabricated. Moreover, through physical characterization methods such as scanning electron microscopy (SEM), atomic force microscopy (AFM), conductive atomic force microscopy (C-AFM) and transmission electron microscopy (TEM), the influencing factors of the crystal structure of the vertical nanosheet were analyzed. This lays the foundation for fabricating high-performance and low-cost RC-VCNFETs devices in the future.

## 1. Introduction

With the evolution of Moore’s law, it becomes more and more difficult to scale down transistor size [1]. The 2 nm technology nodes Samsung, Intel, and TSMC will all have the architecture of gate-all-around field effect transistors (GAAFETs) [2,3,4,5]. The dynamic random-access memory (DRAM) roadmap of the International Roadmap for Devices and Systems (IRDS) 2020 report proposes that the cell transistor structure of DRAM will shift from one of the current mainstream Saddle Fin to the vertical channel transistor (VCT) [6,7,8,9,10,11]. In logic applications, IBM and Samsung jointly proposed vertical-transport FET (VTFET), which achieved a 40 nm contacted gate pitch (CGP) under excellent gate control, which is significantly lower than the 45 nm CGP of the TSMC 3 nm fin field-effect transistor (FinFET) technology node [12,13,14]. This proves that the vertical device has great potential for future device footprint scaling. However, the patterning of the vertical transistor channel mainly relies on advanced lithography and etching, which are accompanied by large process fluctuations. Implementing the self-alignment technology in lateral devices for gates and device channels is also challenging. As a result, there are large fluctuations in the vertical device’s performance.

Based on the advanced Si/SiGe/Si epitaxy and SiGe selective etching process, self-aligned vertical sandwich FET (VSAFET) and vertical C-shaped-channel nanosheet FET (VCNFET) devices were proposed successively [15,16,17,18,19]. In this way, gate and source/drain self-alignment was realized. At the same time, a device with a strong gate control ability and a large driving current was prepared. However, the above devices all use expensive epitaxial processes. At the same time, introducing SiGe epitaxy at the front-end-of-line (FEOL) of the process flow caused the problem of Ge contamination. Controlling the contamination of Ge during mass production significantly reduces the versatility of related equipment, making it difficult to process other Ge-free devices, and thus increasing the cost of mass production.

This paper proposes a recrystallization-based vertical C-shaped-channel nanosheet field effect transistor (RC-VCNFET) and process integration method. The entire process of the device did not involve the Ge element, avoiding the use of expensive epitaxial processes. This method utilizes the principle of laser annealing and recrystallization of the a-Si. A high-quality recrystallized C-shaped Si channel was fabricated at the nanometer scale, by optimizing the laser annealing energy and other process parameters. Through physical characterization methods, it was found that the lattice structure of Si in the channel is very close to a single-crystal structure. This result can provide a foundation for the future fabrication of low-cost, high-mobility vertical channel devices.

## 2. Materials and Methods

**Figure 1:**
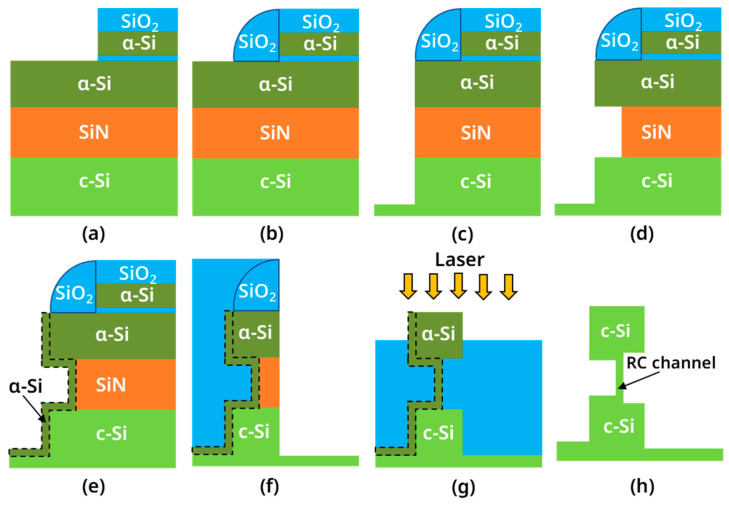
Schematic diagram of the key process steps for the RC-VCNFETs: (**a**) OSO HM formation; (**b**) oxide spacer formation; (**c**) a-Si/SiN/c-Si etching; (**d**) SiN etching; (**e**) Si cap depsition; (**f**) inner a-Si/SiN/c-Si etching; (**g**) STI recess and laser annealing; (**h**) RC channel release.

Figure 1a–h illustrates the main process flow for forming the recrystallized vertical nanosheet. First, 80 nm SiN was deposited on a silicon substrate via plasma-enhanced chemical vapor deposition (PECVD, AMAT Producer S PECVD, Applied Materials, Santa Clara, CA, USA), and 180 nm a-Si was deposited at 580 °C via rapid thermal chemical vapor deposition (RTCVD, Centura, Applied Materials, Santa Clara, CA, USA). Next, 10 nm SiO_2_, 300 nm a-Si and 300 nm SiO_2_ stacks (abbreviated as OSO stacks) were sequentially deposited via PECVD. Moreover, the OSO hard mask (OSO HM) structure in Figure 1a was formed via lithography and etching. Then, silicon oxide sidewalls were formed sequentially, depositing an oxide and anisotropic etching oxide, as shown in Figure 1b. Then, using the silicon oxide as a hard mask, the a-Si/SiN/c-Si stacks were etched through an anisotropic RIE process to form the structure shown in Figure 1c. Then, SiN was etched isotropically by 160 °C H_3_PO_4_ to form a C-shaped-cavity structure, as shown in Figure 1d. Next, a 20 nm thick Si cap was grown via RTCVD. When growing the Si cap, a diluted buffered oxide etchant (dBOE) was used to remove the natural oxide layer on the c-Si surface in the C-shaped-cavity. After the Si cap growth was completed, the Si cap on the OSO HM and oxide spacer was removed, forming the structure in Figure 1e via RIE. Then a high-aspect-ratio-process(HARP) oxide was deposited via PECVD, and the wafer was polished via a chemical mechanical planarization (CMP, FRX200, Ebara, Tokyo, Japan) process until the mandrel in the OSO HM was exposed. Moreover, the a-Si was removed using a high-selectivity TMAH wet etch. Next, 10 nm silicon oxide CESL was etched via RIE. Next, using silicon oxide as a mask, the inner a-Si/SiN/c-Si stacks were etched via RIE to form the structure shown in Figure 1f. Next, the remaining SiN in the device cavity was removed using 160 °C H_3_PO_4_. Then, the HARP oxide was deposited again and CMP was performed on it so that the height of the silicon oxide was about 20 nm from the top of the a-Si. Then, the diluted hydrofluoric acid solution (dHF) was used for the oxide recess process so that the surface of the HARP oxide was lowered to the position shown in Figure 1g. At this point, about half of the top a-Si of the RC-VCNFET was exposed. Subsequently, four groups of Nd:YLF pulsed lasers with different energy densities were used to irradiate the wafer’s surface (the laser annealing equipment was developed by the Institute of Microelectronics, Chinese Academy of Sciences, the laser’s wavelength is 527 nm, the pulse width is 200 ns, and the frequency is 200 Hz). At this time, the a-Si began to recrystallize under laser light irradiation. Finally, we continued to etch the HARP oxide through the STI recess process to release the RC channel. In the next experiment, if RC-VCNFET devices need to be fabricated, process steps such as the gate stacks formation and subsequent BEOL should be carried out.

**Figure 2:**
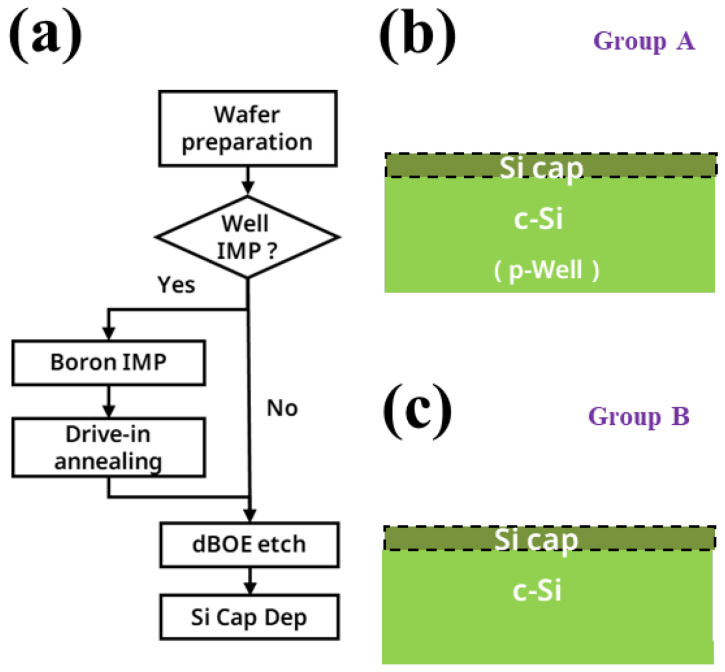
(**a**) Flow diagram of the laser annealing experiment; schematic diagram of (**b**) group A wafer with p-well after Si cap growth; (**c**) group B wafer without p-well after Si cap growth.

In addition, we also conducted a short loop of the Si cap growth module and the flow chart is shown in Figure 2a. We have reported the experiment of Group A, in another work. In that experiment, the blank wafers used all had high-energy boron ion implantation. Therefore, we conducted new experiments on Group B, and the blank wafers in this group of experiments did not have a p-well. The following are the experimental steps. Firstly, a pre-clean step was performed on two groups of silicon wafers, one without dBOE etching and the other with 60 s dBOE etching. These two groups of wafers were respectively named wafer(B-1) and wafer(B-2). Subsequently, these wafers were immediately loaded into the chamber of RTCVD, thereby reducing the formation of the natural oxide layer on the surface of the wafer. Next, a 40 nm-thick Si cap was grown on the surface of the wafer at 580 °C. 

Scanning electron microscopy (SEM, S-5500, Hitachi, Tokyo, Japan) was used to observe the topography of the surface and cross-section of the sample, thereby measuring the film thickness and etching depth. Atomic force microscopy (AFM, Dimension Icon, Bruker, Karlsruhe, Germany) was used to evaluate the film surface’s roughness. Conductive atomic force microscopy (C-AFM, Dimension Icon, Bruker, Karlsruhe, Germany) was used to characterize the conductivity of the nanosheet. Transmission electron microscopy (TEM, FEI Talos F200, Hillsboro, OR, USA) was used to characterize the device’s component dimensions and crystal structure. Energy-dispersive spectroscopy (EDS) was used to determine the distribution of various elements in the device. Nano-beam diffraction (NBD) was used to analyze the crystal structure of the channel

## 3. Results and Discussion

### 3.1. Structural Analysis of the Si Cap Film Based on RTCVD

**Figure 3:**
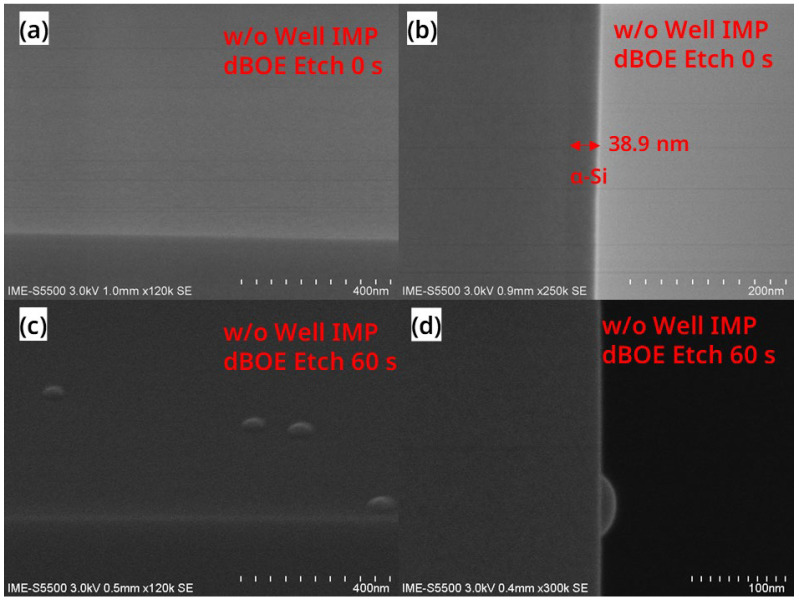
SEM images of (**a**) the surface and (**b**) the cross-sectional view of the blank wafer after the “dBOE cleaning 0 s + Si cap growth” process; SEM images of (**c**) the surface and a (**d**) cross-sectional view of the wafer after the “dBOE cleaning 60 s + Si cap growth” process.

Figure 3a is a SEM image of the sample surface of wafer(B-1) after the “dBOE cleaning 0 s + Si cap growth” step, and it can be seen that the wafer has a very smooth surface. In Figure 3b, there is a layer of a-Si film with a thickness of 38.9 nm on the surface of wafer(B-1), and the contrast between the a-Si film and the single-crystal Si of the substrate is different, proving that the a-Si/c-Si interface exists. This result may be due to the natural oxide layer on the wafer having a blocking effect on the Si (100) crystal plane, suppressing the regular arrangement of Si atoms during the growth of the Si cap.

Figure 3c is a SEM image of the wafer(B-2) surface after the “dBOE cleaning 60 s + Si cap growth” step, and Figure 3d is its cross-sectional SEM image. In Figure 3d, it can be seen that the interface of a-Si/c-Si is not visible after 60 s of BOE cleaning. This result indicates that the Si (100) crystal surface could act as a seed layer. Meanwhile, as the RTCVD chamber was designed for the deposition of a-Si and poly-Si thin films, a small number of particles in the equipment may cause some hillock-like defects such as those in Figure 3c during the growth of the Si cap.

**Figure 4:**
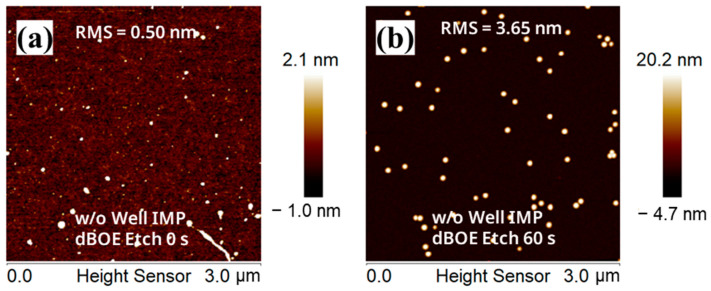
AFM images of the surface of (**a**) the blank wafer after the “dBOE cleaning 0 s + Si cap growth” process and (**b**) the blank wafer after the “dBOE cleaning 60 s + Si cap growth” process.

In addition, AFM tests were carried out on the wafer(B-1) and wafer(B-2) surfaces. In Figure 4a,b, the root mean square roughness (RMS) of the wafer(B-1) surface is 0.50 nm, and the RMS of the wafer(B-2) surface is 3.65 nm. This result is due to some small bulges on the surface in Figure 4b, increasing the RMS of the entire region. The RMS of the non-bulge area on the wafer(B-2) surface is relatively low. These test results show that the pre-clean step significantly impacts the morphology of the Si cap.

### 3.2. Effect of the Laser Annealing Process on the Nanosheet with the RC Channel

**Figure 5:**
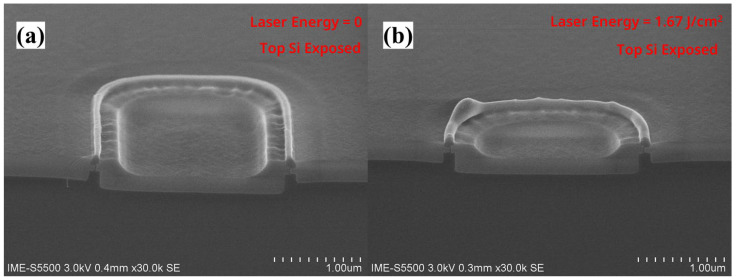
SEM image of the surface of the “exposed vertical nanosheet” (**a**) before and (**b**) after the laser annealing process.

As shown in Figure 1g, we performed a laser annealing experiment on the vertical nanosheet with an “exposed top” structure. Figure 5a is the SEM image of the nanosheet surface before laser annealing, and Figure 5b is the SEM image of the vertical nanosheet surface after laser annealing. The energy density of the laser used was 1.67 J/cm^2^. The above results show that the top silicon of the “exposed vertical nanosheet” changed from having a right-angled surface to a curved surface. This result shows that the top silicon of the nanosheet underwent a recrystallization process of “a-Si (solid)-Si (liquid)-c-Si (solid)” [20,21]. Moreover, it can be seen that the surface morphology of the ring-shaped recrystallized nanosheet was relatively uniform, and no apparent cracks appeared.

**Figure 6:**
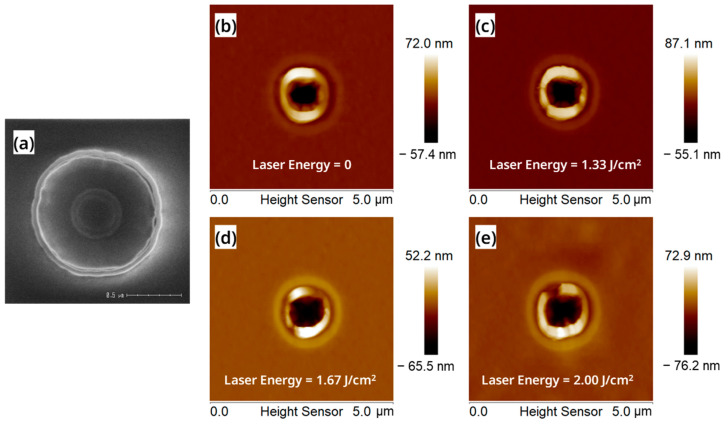
(**a**) Top-view SEM of the surface of the “exposed vertical nanosheet” before the laser annealing process; AFM images of the surface of the “exposed vertical nanosheet” under the laser energy of (**b**) 0 J/cm^2^, (**c**) 1.33 J/cm^2^, (**d**) 1.67 J/cm^2^ and (**e**) 2.00 J/cm^2^.

Next, we also studied the effect of different laser energy densities on the recrystallization process of the vertical nanosheets. Figure 6a is a top-view SEM of the vertical nanosheet before laser annealing. The dishing pits in the center of the circular nanosheet caused by the CMP process can be observed. We used A, B, C, and D, four groups of lasers with different energy densities, to irradiate the “exposed vertical nanosheet” (the laser energy is 0, 1.33, 1.67 and 2.00 J/cm^2^). Obtained after the laser annealing process, the AFM test results of the above four groups of samples are shown in Figure 6b–e. It can be seen from Figure 6b–e that when the laser energy density was 1.67 J/cm^2^, the color difference between the nanosheet top silicon and its surrounding HARP oxide was the smallest, which means that the height difference was the smallest. This indicates that the top silicon of the nanosheet shrunk significantly due to the recrystallization process under this annealing condition. At the same time, when the laser energy density was 2.00 J/cm^2^, the color difference between the top silicon of the nanosheet and the surrounding HARP oxide began to increase, which means that the roughness of the HARP oxide began to increase significantly. This result indicates that the energy of the “2.00 J/cm^2^” laser was too high and began to have an ablation effect on the wafer surface.

**Figure 7:**
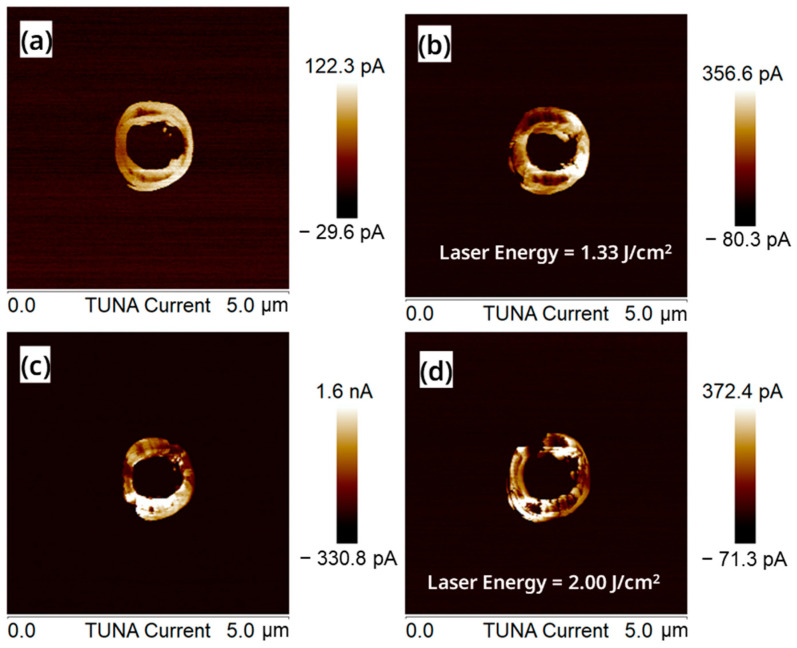
C-AFM images of the surface of the “exposed vertical nanosheet” under the laser energy of (**a**) 0 J/cm^2^, (**b**) 1.33 J/cm^2^, (**c**) 1.67 J/cm^2^ and (**d**) 2.00 J/cm^2^.

In addition, we also carried out a C-AFM test on the four groups of samples, A, B, C and D. With the increase in the laser energy density, the tunneling current of nanosheets first increased and then decreased, as shown in Figure 7a–d. This indicates that the laser with the energy density of 1.67 J/cm^2^ was the most favorable for the recrystallization. Under the condition of 1.67 J/cm^2^, the energy absorbed by a-Si from the laser was enough to melt itself, and the ablation effect caused by high laser energy was avoided.

### 3.3. Crystal Structure Analysis of the Vertical Nanosheet before Laser Annealing

**Figure 8:**
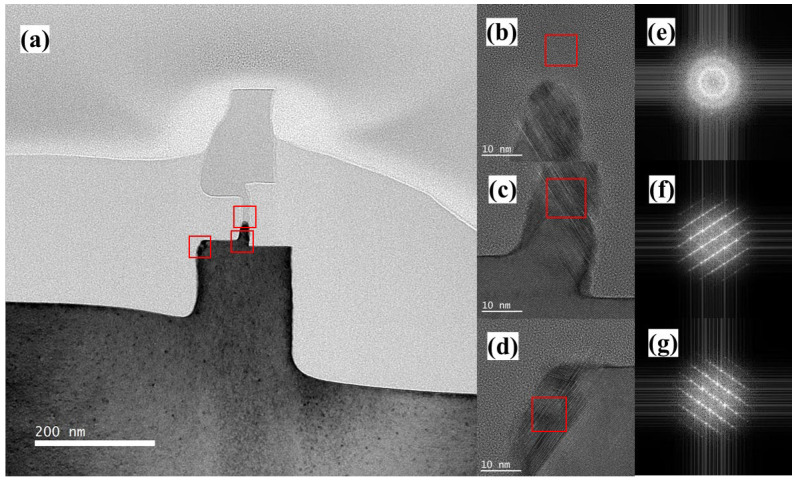
(**a**) TEM image (the red boxs refer to the area of HRTEM images), (**b**–**d**) HRTEM images (the red boxs refer to the area for FFT images), and (**e**–**g**) FFT images of the cross-section of the “exposed vertical nanosheet” before the laser annealing process.

Next, we analyzed the changes in the crystal structure of the vertical nanosheet before and after laser annealing by means of TEM. Figure 8a is the TEM test result of the cross-section of the nanosheet before laser annealing. The C-shaped Si cap at the bottom of the channel and the single-crystal Si substrate have a black contrast, which indicates that the Si cap grown on Si (100) by RTCVD was a single-crystal structure. Simultaneously, the top silicon of the vertical nanosheet and the upper half of the C-shaped channel had a light contrast, indicating that the silicon in these regions was amorphous. Using Figure 8a, the Si cap thickness can be measured. The thickness of the Si cap grown on c-Si, SiN and a-Si was 10.4 nm, 13.1 nm and 23.1 nm, respectively. This is a clear deviation from the expected growth thickness. The different growth rates of Si caps on these interfaces were due to the differences in their respective surface chemical reaction rates. Figure 8b–d shows the HRTEM images of the three regions of the nanosheet in Figure 8a. In Figure 8b, the lattice diffraction signal cannot be observed in the upper half of the C-shaped channel, and the FFT image in Figure 8e shows a dispersed circle. These results indicate that the Si cap in this region was amorphous, like the HARP oxide in Figure 8b. The regions in Figure 8c,d are all in black contrast, and both have Si (111) plane-aligned twin dislocations. At the same time, the FFT results in Figure 8f,g also show a diamond-shaped pattern of the Si (110) crystal plane. These results show that the Si cap grown near the Si (100) seed layer had a single-crystal structure, but the annealing process is required to eliminate defects such as twin dislocations.

**Figure 9:**
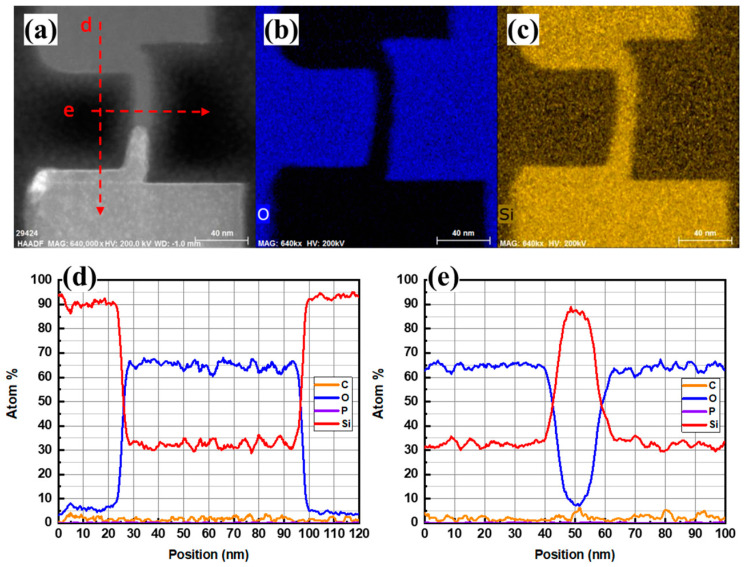
(**a**) HAADF image, (**b**) O element and (**c**) Si element mapping of the cross-section, (**d**) EDS curve of the line scan in the vertical direction, and (**e**) EDS curve of the line scan in the horizontal direction of the “exposed vertical nanosheet” before the laser annealing process.

Figure 9a–c is the HAADF-STEM image of the cross-section of the vertical nanosheet before laser annealing, and the EDS mapping images of O and Si elements, respectively. Figure 9d,e are the EDS line scan results of the red dotted line in Figure 9a. In the HAADF-STEM image, there is an obvious interface between the Si cap layer and the bottom c-Si seed layer before laser annealing, and the twin dislocations in the lower left corner of the C-shaped channel are in bright-white contrast. This shows that although the Si cap film grown by RTCVD could form a structure close to that of a single crystal with the assistance of the c-Si seed layer, the film may still have had some lattice defects. These defects may need to be repaired via a laser annealing process. The EDS line scan results in Figure 9d show that there was no significant oxygen element at the Si cap/c-Si interface, which indicates that the “dBOE 60 s cleaning” process removed the natural oxide layer on the surface of the c-Si seed layer, enabling the growth of the single-crystal Si cap on the seed. According to the curve of the Si element in Figure 9e, the thickness of the Si cap was about 13.4 nm, which is basically consistent with the results in Figure 8a.

### 3.4. Crystal Structure Analysis of the Vertical Nanosheet after Laser Annealing

**Figure 10:**
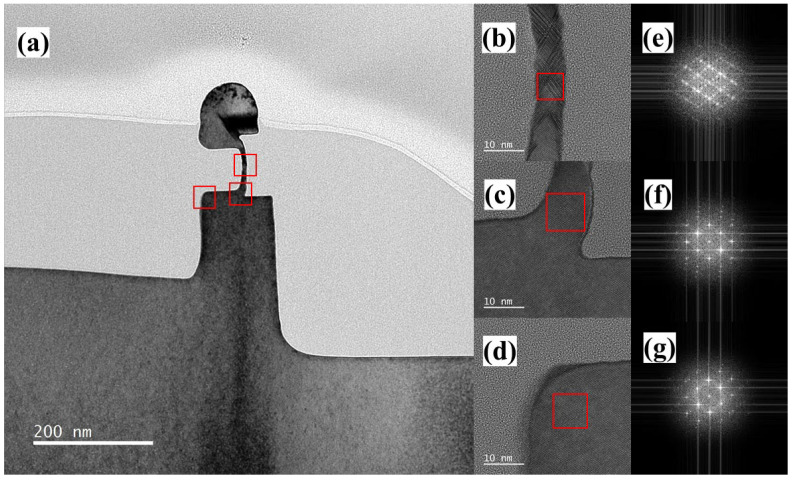
(**a**) TEM image (the red boxs refer to the area of HRTEM images), (**b**–**d**) HRTEM images (the red boxs refer to the area for FFT images), and (**e**–**g**) FFT images of the cross-section of the “exposed vertical nanosheet” after the laser annealing process.

**Figure 11:**
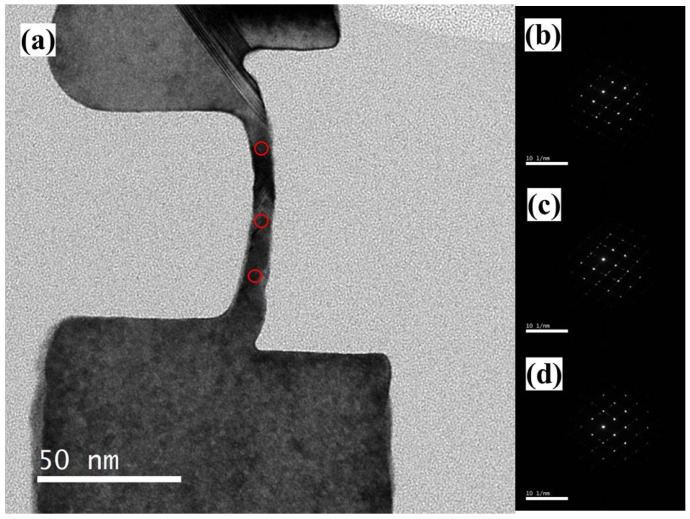
(**a**) TEM image (the red circles refer to the area of NBD images) and (**b**–**d**) NBD images of the cross-section of the “exposed vertical nanosheet” after the laser annealing process.

Figure 10a is the TEM image of the vertical nanosheet after laser annealing. Compared with to sample before laser annealing, the TEM images of the C-shaped channel and the top silicon show the black contrast of the single crystal. In Figure 10b,e, twin dislocations exist in the middle region of the sample channel after annealing. In Figure 10c,d, compared to the samples before annealing, the twin dislocations in these regions of the samples disappeared after annealing, indicating that the laser annealing process can repair these dislocation defects. Next, as shown in Figure 11a–d, the three regions of the nanosheet channel were tested via nanobeam diffraction (NBD), and the spot size of the electron beam was 0.45 nm. The results show that the three regions of the channel all exhibit diffraction patterns of the Si (110) plane index.

**Figure 12:**
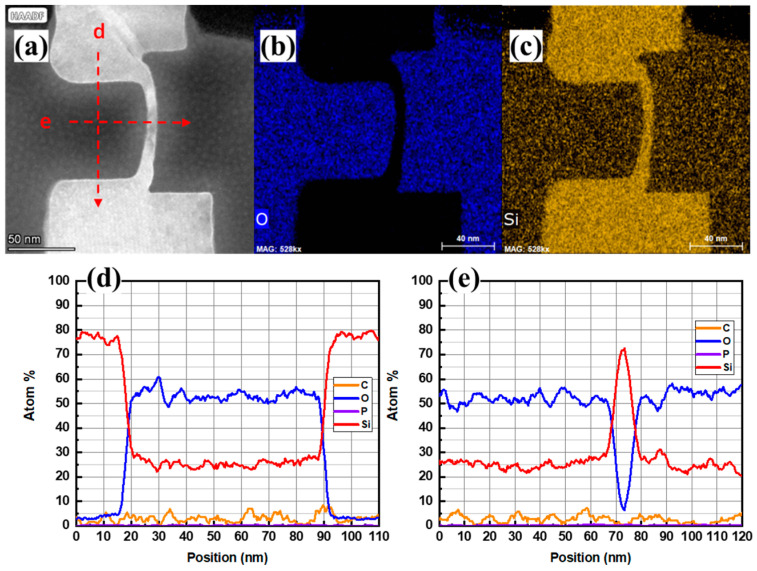
(**a**) HAADF image, (**b**) O element and (**c**) Si element mapping of the cross-section, (**d**) EDS test curve of line scan in vertical direction, and (**e**) EDS test curve of line scan in horizontal direction of the “exposed vertical nanosheet” before the laser annealing process.

Figure 12a–c is the HAADF-STEM image of the cross-section of the vertical nanosheet after the laser annealing process and the EDS mapping images of the O element and the Si element. Figure 12d,e shows the EDS line scan results of the red dotted line in Figure 12a. From the HADDF diagram, the Si cap/c-Si interface of the sample after annealing can be seen. At the same time, it can be seen that the bright spot of the twin dislocations in the lower left corner of the nanosheet disappeared. This shows that the laser annealing process has a good repair effect on lattice defects. In Figure 12b,d, the interface of Si cap/c-Si has no oxygen enrichment. Via the comparison of Figure 12e and Figure 9e, it can be seen that the thickness of the middle channel of the vertical nanosheet with the “exposed top” structure was reduced from 13.4 to 7.0 nm after the laser annealing process. This may be due to the compressive stress exerted by the HARP oxide on the circular vertical nanosheet. During the laser annealing process, the a-Si changed from being in a solid state to a free-flowing liquid Si when the nanosheet absorbed laser energy. At this time, the liquid Si moved upward, the compressive stress on the vertical nanosheet channel began to release, and the surrounding HARP oxide was displaced, which finally reduced the thickness of the nanosheet.

**Figure 13:**
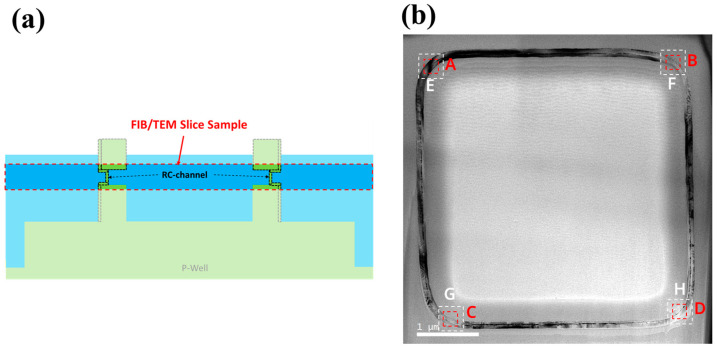
(**a**) Schematic diagram of the FIB sample and (**b**) top-view TEM image of the “exposed vertical nanosheet” after the laser annealing process (the red boxes A–D refer to the area of HRTEM images, and the white boxes E–H refer to the area of HADDF images).

In order to further characterize the recrystallization effect of the laser annealing process on larger-sized devices, we also conducted laser annealing experiments on the “exposed vertical nanosheet” with a size of 4 μm × 4 μm and performed a top-view-TEM test analysis. Figure 13a is a schematic diagram of the structure of the TEM sample preparation area. The red-framed part is the FIB slice sample, and the sample thickness was about 100 nm.

**Figure 14:**
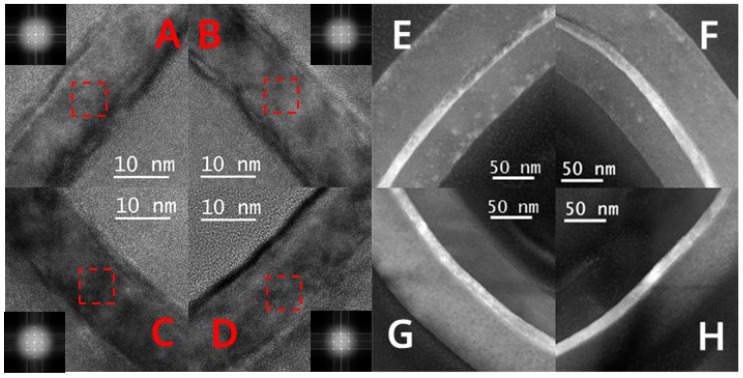
Top view of (**A**–**D**) HRTEM (the red boxs refer to the area for FFT images) and (**E**–**H**) HAADF images of the “exposed vertical nanosheet” after the laser annealing process.

Figure 13b is the TEM test result of the sample’s top view. The channel with a size of 4 μm × 4 μm remained intact and continuous after laser annealing, which proves the uniformity of the recrystallization process of the “exposed vertical nanosheet”. In the HRTEM and FFT images of Figure 14A–D, the channels at the four corners of the sample are all single-crystal structures. In Figure 13e–h, the HAADF images also prove the integrity and continuity of the channel with a size of 4 μm × 4 μm. In Figure 14E–H, the average projected width of the nanosheet channel is about 13.5 nm. This thickness indicates that the RC-VCNFETs device fabricated by the laser annealing process has superior gate control capability.

### 3.5. Electrical Properties of the "Exposed-Top" RC-VCNFET Device

**Figure 15:**
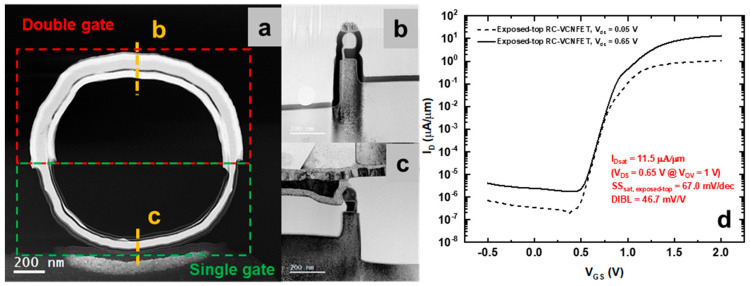
(**a**) The Top View TEM image in dark field, (**b**,**c**) the Cross-section TEM in bright field of the “exposed-top” RC-VCNFET device, and (**d**) the I_D_-V_GS_ curves of the "exposed-top" RC-VCNFET.

Finally, the RC-VCNFET device was fabricated successfully. Figure 15a and Figure 15b,c are the TEM images of the device’s top view and cross-section view, respectively. It can be seen from Figure 15a that the upper half of the ring-shaped RC-VCNFET device is a double-gate device, while the lower half of the ring-shaped device is a single-gate device. Next, the electrical test was performed on the "exposed-top" RC-VCNFETs device, and the results are shown in Figure 15d. The I_on_ of this RC-VCNFET device is 11.5 μA/μm (I_D_ @ V_OV_ = V_G_ − V_T_ = 1 V, V_DS_ = 0.65 V). The SS of the device is 67.0 mV/dec, and the DIBL of the device is 46.7 mV/V. These test results indicate that the performance of the RC-VCNFET needs to be further optimized.

## 4. Conclusions

This paper introduces the effect of pre-cleaning conditions on the surface of the wafer on the Si cap film grown by RTCVD. SEM and AFM results revealed that an amorphous Si cap grew on the single-crystal silicon without the pre-clean step. In addition, the laser annealing process was carried out on the vertical nanosheet with an “exposed top” structure, and the crystal structure of the vertical nanosheet before and after laser annealing was characterized by means of SEM, AFM, C-AFM, TEM and NBD. Finally, the high-quality recrystallized vertical nanosheet structure was successfully fabricated, which laid a certain foundation for the preparation of high-performance and low-cost vertical-channel devices in the future.

## Data Availability

The data presented in this study are available upon request from the corresponding author.

## References

[1] Zhang S. (2020). Review of Modern Field Effect Transistor Technologies for Scaling. J. Phys. Conf. Ser..

[2] Bae G., Bae D.-I., Kang M., Hwang S.M., Kim S.S., Seo B., Kwon T.Y., Lee T.J., Moon C., Choi Y.M. 3 nm GAA Technology Featuring Multi-Bridge-Channel FET for Low Power and High Performance Applications. Proceedings of the 2018 IEEE International Electron Devices Meeting (IEDM).

[3] Song T., Kim H., Rim W., Jung H., Park C., Lee I., Baek S., Jung J. (2022). A 3-Nm Gate-All-Around SRAM Featuring an Adaptive Dual-Bitline and an Adaptive Cell-Power Assist Circuit. IEEE J. Solid-State Circuits.

[4] Huang C.-Y., Dewey G., Mannebach E., Phan A., Morrow P., Rachmady W., Tung I.-C., Thomas N., Alaan U., Paul R. 3-D Self-Aligned Stacked NMOS-on-PMOS Nanoribbon Transistors for Continued Moore’s Law Scaling. Proceedings of the 2020 IEEE International Electron Devices Meeting (IEDM).

[5] Liu M. 1.1 Unleashing the Future of Innovation. Proceedings of the 2021 IEEE International Solid-State Circuits Conference (ISSCC).

[6] Park S.-K. Technology Scaling Challenge and Future Prospects of DRAM and NAND Flash Memory. Proceedings of the 2015 IEEE International Memory Workshop (IMW).

[7] Yang C.-M., Wei C.-K., Chang Y.J., Wu T.-C., Chen H.-P., Lai C.-S. (2016). Suppression of Row Hammer Effect by Doping Profile Modification in Saddle-Fin Array Devices for Sub-30-Nm DRAM Technology. IEEE Trans. Device Mater. Reliab..

[8] Lee S.-W., Kim S.-Y., Hwang K.-M., Jin I.K., Hur J., Kim D.-H., Son J.W., Kim W.-K., Choi Y.-K. (2018). A Comprehensive Study of a Single-Transistor Latch in Vertical Pillar-Type FETs with Asymmetric Source and Drain. IEEE Trans. Electron Devices.

[9] Cho Y.S., Choi P.H., Kim K.H., Park J.M., Hwang Y.S., Hong H.S., Lee K.P., Choi B.D. (2019). Stretched Tunnelling Body Contact Structure for Suppressing the FBE in a Vertical Cell DRAM. Electron. Lett..

[10] Cho Y., Kim H., Jung K., Kim B., Hwang Y., Hong H., Choi B. (2018). Suppression of the Floating-Body Effect of Vertical-Cell DRAM with the Buried Body Engineering Method. IEEE Trans. Electron Devices.

[11] Cho Y., Choi P., Hyeon Y., Song J., Hwang Y., Choi B. (2018). Novel Band-to-Band Tunneling Body Contact (BTBC) Structure to Suppress the Floating-Body Effect in a Vertical-Cell DRAM. IEEE Electron Device Lett..

[12] Jagannathan H., Anderson B., Sohn C.-W., Tsutsui G., Strane J., Xie R., Fan S., Kim K.I., Song S., Sieg S. Vertical-Transport Nanosheet Technology for CMOS Scaling beyond Lateral-Transport Devices. Proceedings of the 2021 IEEE International Electron Devices Meeting (IEDM).

[13] Tsutsui G., Song S., Strane J., Xie R., Qin L., Zhang C., Schmidt D., Fan S., Hong B., Jung Y. Hardware Based Performance Assessment of Vertical-Transport Nanosheet Technology. Proceedings of the IEEE International Electron Devices Meeting.

[14] Chang C.-H., Chang V.S., Pan K.H., Lai K.T., Lu J.H., Ng J.A., Chen C.Y., Wu B.F., Lin C.J., Liang C.S. Critical Process Features Enabling Aggressive Contacted Gate Pitch Scaling for 3nm CMOS Technology and Beyond. Proceedings of the 2022 International Electron Devices Meeting (IEDM).

[15] Yin X., Zhang Y., Zhu H., Wang G.L., Li J.J., Du A.Y., Li C., Zhao L.H., Huang W.X., Yang H. (2019). Vertical Sandwich Gate-All-around Field-Effect Transistors with Self-Aligned High-k Metal Gates and Small Effective-Gate-Length Variation. IEEE Electron Device Lett..

[16] Zhang Y., Ai X., Yin X., Zhu H., Yang H., Wang G.L., Li J.J., Du A.Y., Li C., Huang W.X. (2021). Vertical Sandwich GAA FETs with Self-Aligned High-k Metal Gate Made by Quasi Atomic Layer Etching Process. IEEE Trans. Electron Devices.

[17] Li C., Zhu H., Zhang Y., Wang Q., Yin X., Li J., Wang G., Kong Z., Ai X., Xie L. (2021). First Demonstration of Novel Vertical Gate-All-around Field-Effect-Transistors Featured by Self-Aligned and Replaced High-κ Metal Gates. Nano Lett..

[18] Xiao Z.R., Wang Q., Zhu H.L., Chen Z., Zhang Y.K., Li J.J., Zhou N., Gao J.F., Ai X.Z., Lu S.S. (2022). Vertical C-Shaped-Channel Nanosheet FETs Featured with Precise Control of Both Channel-Thickness and Gate-Length. IEEE Electron Device Lett..

[19] Xiao Z.R., Zhu H.L., Wang Q., Chen Z., Liu Z.Y., Zhang Y.K., Yan Z.J., Shi Y.F., Zhou N., Li J.J. (2023). Vertical N-Type and P-Type Nanosheet FETs with C-Shaped Channel. IEEE Trans. Electron Devices.

[20] Liu Y.-W., Hu H.-W., Hsieh P.-Y., Chung H.-T., Chang S.-J., Liu J.-H., Huang P.-T., Yang C.-C., Shen C.-H., Shieh J.-M. (2021). Single-Crystal Islands (SCI) for Monolithic 3-D and Back-End-of-Line FinFET Circuits. IEEE Trans. Electron Devices.

[21] Son Y.-I., Shin J. (2022). Numerical Study on the Laser Annealing of Silicon Used in Advanced V-NAND Device. Materials.

